# Antioxidants in Food: The Significance of Characterisation, Identification, Chemical and Biological Assays in Determining the Role of Antioxidants in Food

**DOI:** 10.3390/foods6080068

**Published:** 2017-08-14

**Authors:** Barry J. Parsons

**Affiliations:** School of Clinical and Applied Sciences, Leeds Beckett University, Leeds LS1 3HE, UK; b.parsons@leedsbeckett.ac.uk

There is a vast research literature on the antioxidant activity in food. Figure 1 shows the linear growth in the number of papers per year as found in the Web of Science database using the search item “food AND antioxidant”. In 2016, the number was 3304 papers on this topic. Most of these determine total antioxidant activity or capacity and an ever-increasing number analyze and identify the individual antioxidant molecules. Combined with these measurements, a number of antioxidant assays, such as DPPH, ABTS, ORAC, FRAP and TEAC, are commonly used (Appendix A). In a few cases, biological assays, typically involving cell culture, also provide useful information on the effectiveness of antioxidants.

In the case of foods seen as a natural mix of antioxidants, the highly cited paper by Floegel et al., produced an extensive correlation of the antioxidant capacities of 50 fruit, vegetable and beverages using the ABTS, DPPH and ORAC techniques with their total phenolic and flavanoid contents [1]. It was suggested that the ABTS method may be more useful than the DPPH method for measuring antioxidant capacity in a variety of foods.

Foods contain many antioxidants, some of which are more common than others. Flavonoids, for example, are often found in significant concentrations in most natural foods. Such individual antioxidant molecules react at different rates of reaction with free radicals and reactive oxidative species ROS) and, hence, have the potential to avoid the damaging effects of these reactive species on cellular components, particularly DNA. Antioxidant assays all purport to measure total antioxidant activity but yet different assays often do not correlate when measuring the same mixture of antioxidant molecules. Many assays are now informed by precise identification of the individual antioxidant molecules. In addition, there is much data now available on the reactivities of such molecules with specific free radicals and ROS. In principle, therefore, it is possible to reconcile the correlation (or lack of) of antioxidant mixtures. 

In this Special Issue, the original papers and review provide further insights into the correlation of characterisation, identification, chemical, biological and other assays of food-based antioxidant mixtures.

In the study by Zeb [2], using water, methanol and methanol/water extracts from melon seeds, nine phenolic compounds were identified using HPLC and the Folin Ciocalteau method for total phenolic content. The major component was caffeic acid together with significant contributions from gallic acid and catechin. Diphenylpicrylhydrazine (DPPH) radicals were used to determine antioxidant activity. The study demonstrated that the type of extraction solvent had a large effect on the efficiency of extraction of phenolics. Those containing water resulted in efficient extraction. There was also a broad correlation of antioxidant activity with total phenolic content.

De Taeye and Kankolongo, used online HPLC/TEAC and high resolution MS techniques to follow the heat-induced degradation of procyanidin, the major flavanol-3-ol trimer extracted from cocoa [3]. Epimerization was the major process. Oxidized dimers were also produced whose antioxidant activities were similar to procyanidin itself.

Proestos and Varzakas extracted antioxidants from 8 aromatic plants found in Greece and measured their activities using the DPPH method [4]. Catechin, quercetin and rutin were found to be the major flavanoids whereas caffeic acid and ferrulic acid were the common phenolic acid components. They found a 35 fold difference in the range of activities when using ascorbic acid and butylated hydroxytoluene as standards. 

In the review by Durazzo, many factors affecting the antioxidant activities of foods were considered [5]. Thus, the efficiencies of the various methods of chemical extraction methods were discussed in terms of the type of extraction method, time of extraction and temperature. The highest values were usually found in acidic MeOH/H_2_O solutions. Other methods such as alkaline and acidic hydrolysis as well as enzymatic methods were also highlighted. Durazzo also drew attention to the antioxidant activities of non-extractable antioxidants such as tannins, phenolic acids and hydroxycinnamic acids which often lead to an underestimate of total antioxidant activity. Indeed, some studies were found to have higher antioxidant activities in the non-extractable residues. Equally significant was the effect of processing on antioxidant activities. 

Durazzo also reviewed the status of studies on the antioxidant capacities of the wide range of pure antioxidant compounds often used as a basis for understanding the overall antioxidant activities of extracts and highlighted studies in which a synergistic effect was observed whereby the overall activity was larger than predicted from studies on the separate standards.

Consistent with the theme of this Special Issue, the contributed papers confirm the steadily-growing interest in food and antioxidants. These papers demonstrate not only the ever more sophisticated analytical techniques applied to identify individual antioxidant molecules as components of complex food extracts but also the drive to correlate overall antioxidant capacity of a food with the individual activities of the component antioxidant molecules.

There are many antioxidant activity assays in general use, most of which can be classified as either electron transfer (ET) or hydrogen atom transfer (HAT) mechanisms with ABTS and DPPH the most well used. In all methods, a kinetic competition is set up between the oxidizing agent (ABTS cation radical or DPPH radical) and reducing agents (the food antioxidant mixtures or individual components therein including standards such as Trolox or ascorbic acid). After a limited incubation, typically 6–10 min, the depletion of the oxidising agent is determined. The extent of depletion in the sample mixture can then be compared to a standard such as Trolox. The assumption is that at the end of the incubation period, the measurements can be used to determine the effectiveness of the food antioxidant(s) or components. However, this assumes a straightforward kinetic competition and it is likely that in some cases, this is not valid. There are very few kinetic studies on the reactions of the ABTS cation radical or the DPPH radical with pure food antioxidant components such as quercetin and catechin. Some such studies show that the reaction mechanisms are often complex and need further interpretation [6,7]. For example, 40 μM ABTS cation radical was found to react with 10 μM Trolox within 10 seconds of mixing with approximately two ABTS radicals being required for each Trolox molecule [7]. In the case of t-tertbtulyphenol, however, the reaction required more than 500 s to go to completion with four ABTS radicals being required . In the same study, quercetin produced a bi-exponential decay with lifetimes of about 3 and 200 s. With 1,4-dihydroxybenzene, a complex decay was observed over 700 s showing a significant recovery in the initial depletion of the ABTS cation radical. Factors which influence the mechanism, particularly in ET, include the number of aromatic hydroxyl groups in the antioxidant and also the possibility of equilibrium when the reduction potentials of the redox pair are sufficiently close. In view of the importance and growing interest in the area of food and antioxidants, such focused individual studies on the major antioxidant components of foods deserve a more detailed kinetic and mechanistic approach. This would make the current massive interest in correlations of antioxidant capacity with antioxidant contents of foods more accurately understood. 

## Figures and Tables

**Figure 1:**
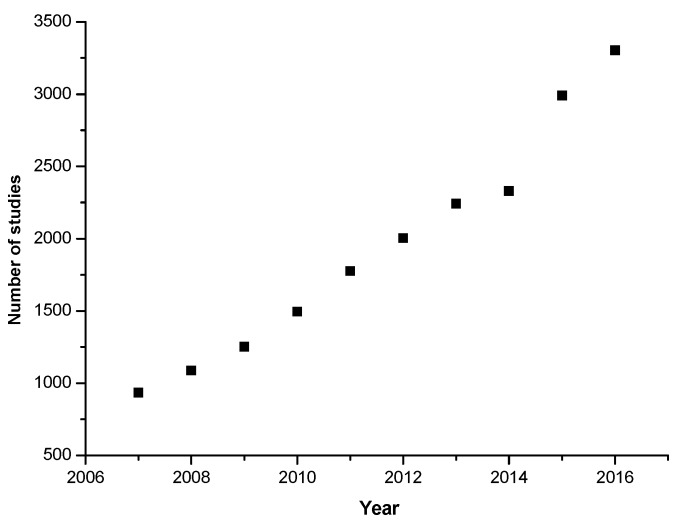
Growth in the number of food and antioxidant studies, from 2007 to 2016.

**Figure A1:**
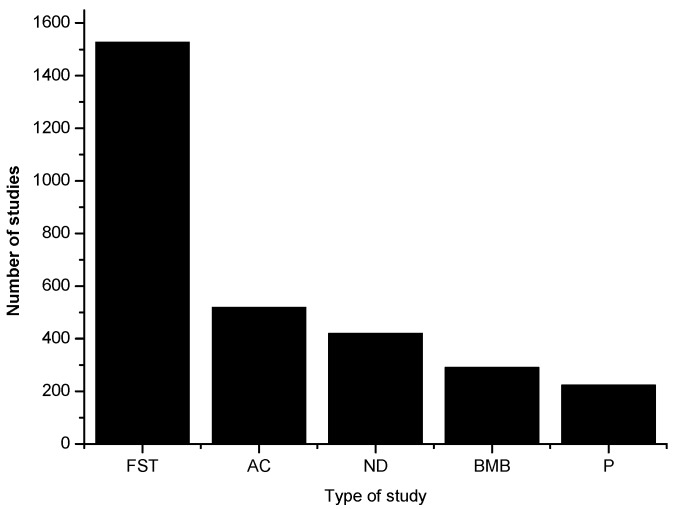
Number of studies in 2016 by discipline (FST, Food Science and Technology; AC, Applied Chemistry; ND, Nutrition and Dietetics; BMB, Biochemistry and Molecular Biology; P, Pharmacology).

**Figure A2:**
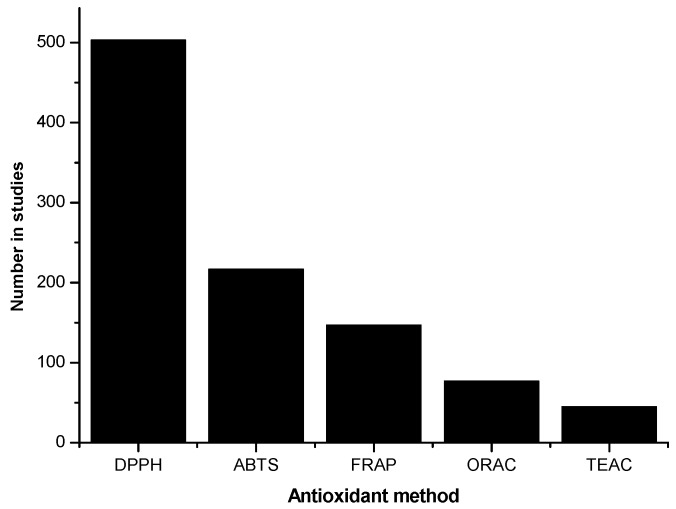
Number of studies in 2016 identifying antioxidant activity method.
